# Low-Temperature Molten Salts Synthesis: CsPbBr_3_ Nanocrystals with High Photoluminescence Emission Buried in Mesoporous SiO_2_

**DOI:** 10.1021/acsenergylett.1c00052

**Published:** 2021-02-11

**Authors:** Mai Ngoc An, Sungwook Park, Rosaria Brescia, Marat Lutfullin, Lutfan Sinatra, Osman M. Bakr, Luca De Trizio, Liberato Manna

**Affiliations:** † Dipartimento di Chimica e Chimica Industriale, Università degli Studi di Genova, Via Dodecaneso 31, 16146 Genova, Italy; ^‡^Nanochemistry Department and ^∥^Electron Microscopy Facility, Istituto Italiano di Tecnologia, Via Morego 30, 16163 Genova, Italy; § Department of Energy Science and Center for Artificial Atoms, Sungkyunkwan University, Suwon, 16419, Republic of Korea; ⊥ Quantum Solutions, 1 Venture Road, Science Park, Southampton, SO16 7NP. U.K. (www.qdot.inc); # Division of Physical Sciences and Engineering, King Abdullah University of Science and Technology (KAUST), Thuwal, 23955-6900, Saudi Arabia

## Abstract

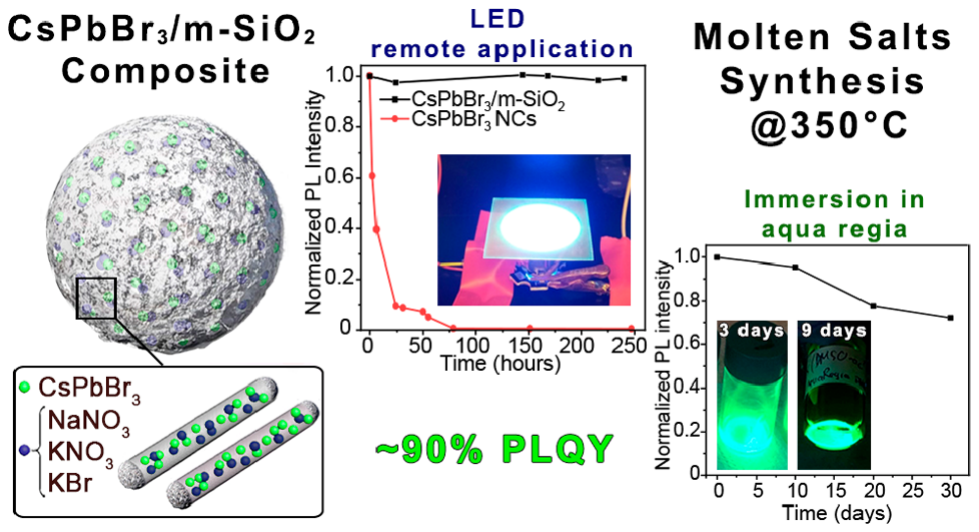

Using mesoporous SiO_2_ to encapsulate CsPbBr_3_ nanocrystals is one of the best strategies to exploit such materials in devices. However, the CsPbBr_3_/SiO_2_ composites produced so far do not exhibit strong photoluminescence emission and, simultaneously, high stability against heat and water. We demonstrate a molten-salts-based approach delivering CsPbBr_3_/mesoporous-SiO_2_ composites with high PLQY (89 ± 10%) and high stability against heat, water, and aqua regia. The molten salts enable the formation of perovskite nanocrystals and other inorganic salts (KNO_3_–NaNO_3_–KBr) inside silica and the sealing of SiO_2_ pores at temperatures as low as 350 °C, representing an important technological advancement (analogous sealing was observed only above 700 °C in previous reports). Our CsPbBr_3_/mesoporous-SiO_2_ composites are attractive for different applications: as a proof-of-concept, we prepared a white-light emitting diode exhibiting a correlated color temperature of 7692K. Our composites are also stable after immersion in saline water at high temperatures (a typical underground environment of oil wells), therefore holding promise as oil tracers.

Nanocrystals (NCs) of lead halide perovskites (LHPs), with the chemical formula of APbX_3_ (A = CH_3_NH_3_, HC(NH)NH_2_, Cs and X = Cl, Br, I) have optimal optical properties, which include a high photoluminescence quantum yield (PLQY) and high color purity (narrow PL emission), making them promising candidates for different optoelectronic applications such as light emitting diodes (LEDs), displays, radiation detectors, and solar concentrators.^1−8^ However, the effective implementation of these NCs in industrial manufacturing processes is limited by their poor stability, which leads to their degradation when they are exposed to humidity, high temperature, and photoirradiation.^9−12^

In order to solve this issue, in recent years different strategies aimed at protecting LHP NCs have been devised, with the most promising ones being their encapsulation in polymers,^13−17^ inorganic matrixes^18,19^ (including metal oxide, e.g., SiO_2_, TiO_2_, Al_2_O_3_^20−28^ and metal halide^29^) or hybrid compounds (e.g., metal–organic frameworks, MOFs).^30−32^ The reported LHP/polymer nanocomposites are characterized by a high PLQY and good moisture/water resistance but a weak thermal resistance.^15,16^ MOFs and metal halides can provide thermal and photostability, but they do not offer protection against water.^29−31^ On the other hand, metal oxides, thanks to their robustness, have the potential to ensure both thermal and water stability to LHPs, while preserving their high PL emission.^20,21^

Among the different metal oxides, mesoporous silica (m-SiO_2_) is one of the best candidates for the encapsulation of LHPs, for the following reasons: (i) it is nontoxic, earth-abundant, and cheap; (ii) it has a high chemical and thermal stability; (iii) its surface can be easily functionalized (to make it hydrophilic or hydrophobic); and (iv) the size of the pores can be finely tuned (from 2 to 50 nm).^33−37^ To date, m-SiO_2_ has been successfully employed as a matrix to grow LHPs NCs in its pores, following various approaches.^22−28^ The composites obtained at low temperatures (maximum 180 °C) via wet chemistry approaches (i.e., colloidal synthesis and impregnation) exhibit a modest PL emission (with the maximum PLQY achieved being 68%),^22^ which can be tuned by varying the size of the SiO_2_ pores,^23,25,38^ and decent stability against photon irradiation and temperature, but a poor stability against water or polar solvents (i.e., LHP NCs inside the m-SiO_2_ pores dissolve when the composites are exposed to water or polar solvents).^23−26,28^ Conversely, the solid-state synthesis, carried out at much higher temperatures (700 °C), recently developed by Zhang et al., delivers LHP/m-SiO_2_ composites with the concomitant sealing of the pores and, thus, featuring a very high stability against water and even acid treatment.^27^ However, the high temperatures employed in this process lead also to partial merging of the SiO_2_ particles and, thus, to the formation of bulk-like aggregates, limiting the PLQY of the final product (63% maximum) and the possible use of such materials in practical devices. Overall, the LHP/m-SiO_2_ composites reported so far do not meet the requirements to be used as phosphors in optoelectronic devices, such as displays, light-emitting diodes, high-energy radiation detectors, and solar concentrators,^5,39^ or as tracers/emitters in fields of technology characterized by harsh conditions, such as bioimaging for clinical purposes^40^ or crude oil extraction.^41^ In such applications, a high PLQY and, at the same time, a high stability in harsh conditions, including high salinity, temperature, and low pH, are essential.

In order to tackle this challenge, in this work, we have devised a new solvent-free synthesis approach to prepare strongly emissive and highly stable CsPbBr_3_/m-SiO_2_ composites. Our synthesis protocol is based on the use of molten salts, that is, a mixture of inorganic salts (KNO_3_, KBr and NaNO_3_ in the present case), as the reaction medium. The use of molten salts for the synthesis of nanostructures has emerged in the last years as an important complementary route to conventional liquid phase approaches: depending on the composition of the mixture of salts, the melting temperature (i.e., the operational temperature) can be tuned from ∼ 100 °C to over 1000 °C, enabling the synthesis of a broad range of different inorganic NC materials.^42−45^ In our specific case, the use of molten salts enables the formation of CsPbBr_3_/m-SiO_2_ composites in air at relatively mild temperatures (∼ 350 °C) and, for specific molten salt compositions, the sealing of the pores of the m-SiO_2_ particles (Scheme 1).

**Scheme 1:**
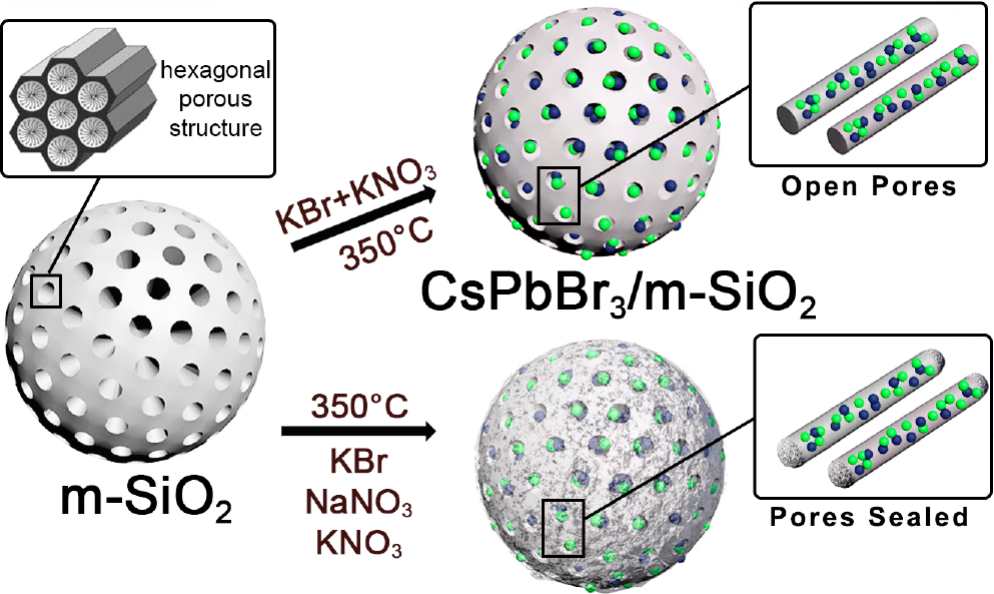
Preparation of CsPbBr_3_/m-SiO_2_ Composites Using the Molten Salts Synthesis Approach and Employing Different Salts Combinations

The final products feature a strong PL emission (PLQY 89 ± 10%) and are highly stable against temperature, fully retaining the initial PL intensity after 3 h at 180 °C or after immersion in water for 30 days and even surviving when immersed in aqua regia (a mixture of HCl and HNO_3_) for at least one month. The high PLQY and high stability of our composites make them optimal candidates for many different applications. We demonstrate here that they can be used as down converting material for a white LED capable of generating a white light with Commission Internationale de l’Eclairage (CIE) color coordinates of (0.2985, 0.3076) and a correlated color temperature (CCT) of 7692 K. Also, our composites retain their luminescence after being exposed to very harsh conditions of saline water (a mixture of NaCl, CaCl_2_, MgCl_2_, Na_2_SO_4_, and NaHCO_3_) and high temperature (90 °C) for 24 h. These conditions are essentially those of crude oil extraction wells, suggesting that our composites can be potential candidates as tracers for the oil-extraction industry.

In a typical synthesis, CsBr and PbBr_2_ (i.e., the perovskite precursors) are mixed with a ternary mixture of molten salts, namely KNO_3_:NaNO_3_:KBr in a 10:5:5 mmol ratio, and m-SiO_2_ particles, and heated up to 350 °C under air in a furnace for 60 min (Scheme 1). The powder, obtained after cleaning the product with dimethyl sulfoxide, features a bright PL emission peaked at 520 nm with a full width at half-maximum of 21.5 nm (99.18 meV) and a PLQY as high as 89 ± 10%, obtained by measuring the sample dispersed in DI water (Figure 1a and 2). The X-ray diffraction (XRD) pattern of the sample is characterized by the presence of peaks ascribable to the orthorhombic CsPbBr_3_ (ICSD 98-009-7851), KBr, NaNO_3_, and KNO_3_ phases (Figure 1d). A broad peak ranging from 15° to 35° is also present in the XRD pattern and is ascribed to the amorphous SiO_2_ matrix (Figure S1a of the Supporting Information). Given the high solubility in polar solvents of all the inorganic salts employed here, the residual salts detected by XRD analysis must have been encapsulated in the pores of m-SiO_2_ particles together with the LHP NCs.

**Figure 1:**
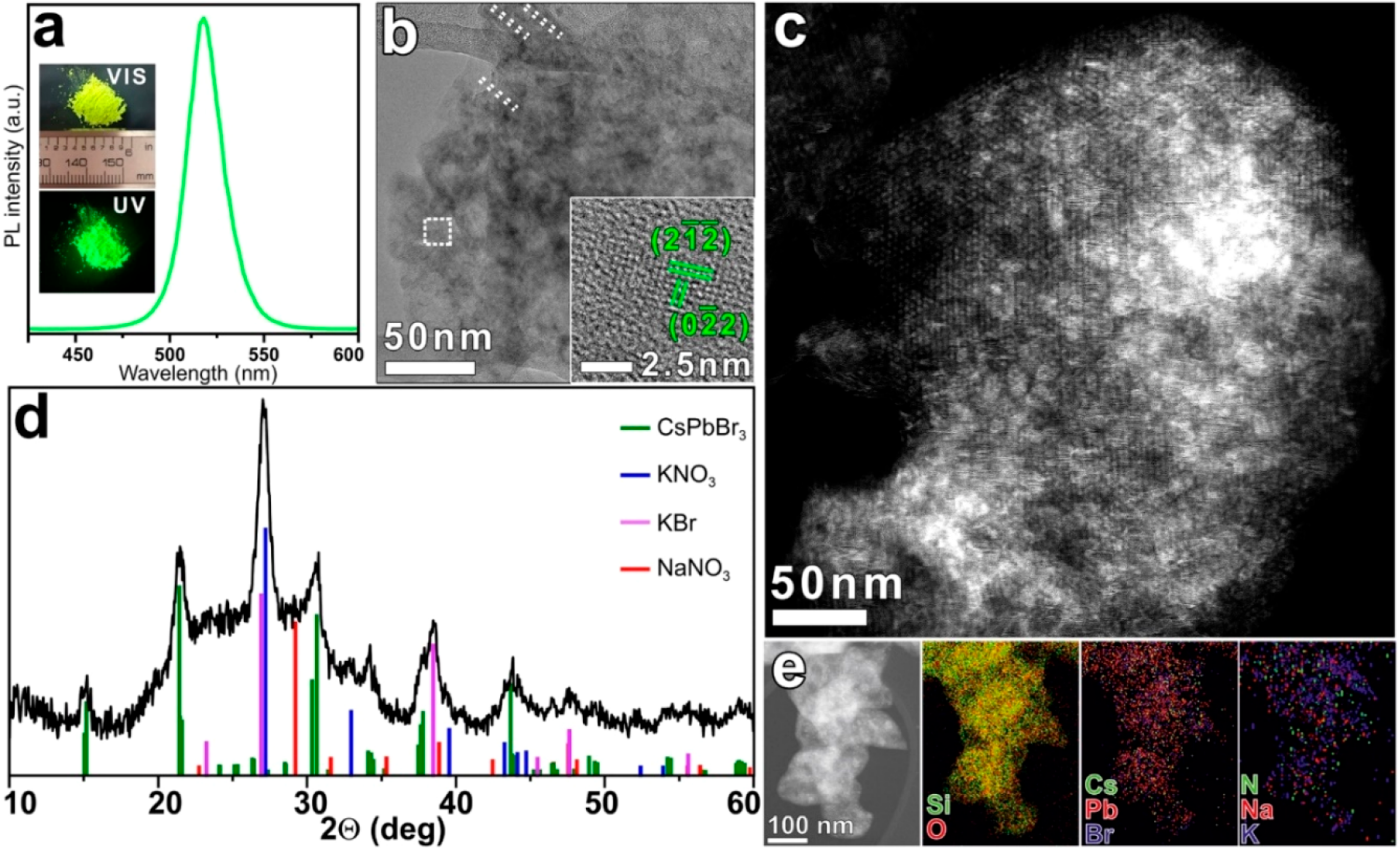
(a) PL spectrum of CsPbBr_3_/m-SiO_2_ composite and (inset) photographs of the sample under visible and UV light (345 nm). (b) BF-TEM images of the CsPbBr_3_/m-SiO_2_ composite, in which it is possible to visualize residual ∼3 nm diameter channels (dashed lines) and higher-Z (darker) particles inside. (Inset) HRTEM image of a CsPbBr_3_ NC embedded in amorphous silica found in the square area highlighted in (b), with lattice planes indexed according to the orthorhombic CsPbBr_3_ phase (ICSD 98-009-7851). (c) HAADF-STEM image of the CsPbBr_3_/m-SiO_2_ composite where it is possible to appreciate the partial collapse of the mesoporous structure. (d) XRD pattern of the CsPbBr_3_/m-SiO_2_ composite together with the bulk reflections of CsPbBr_3_ (ICSD 98-009-7851), KBr (ICSD 98-005-3826), KNO_3_ (ICSD 98-003-6113) and NaNO_3_ (98-001-5333). (e) HAADF-STEM images of the CsPbBr_3_/m-SiO_2_ composite and the corresponding EDS elemental maps.

**Figure 2:**
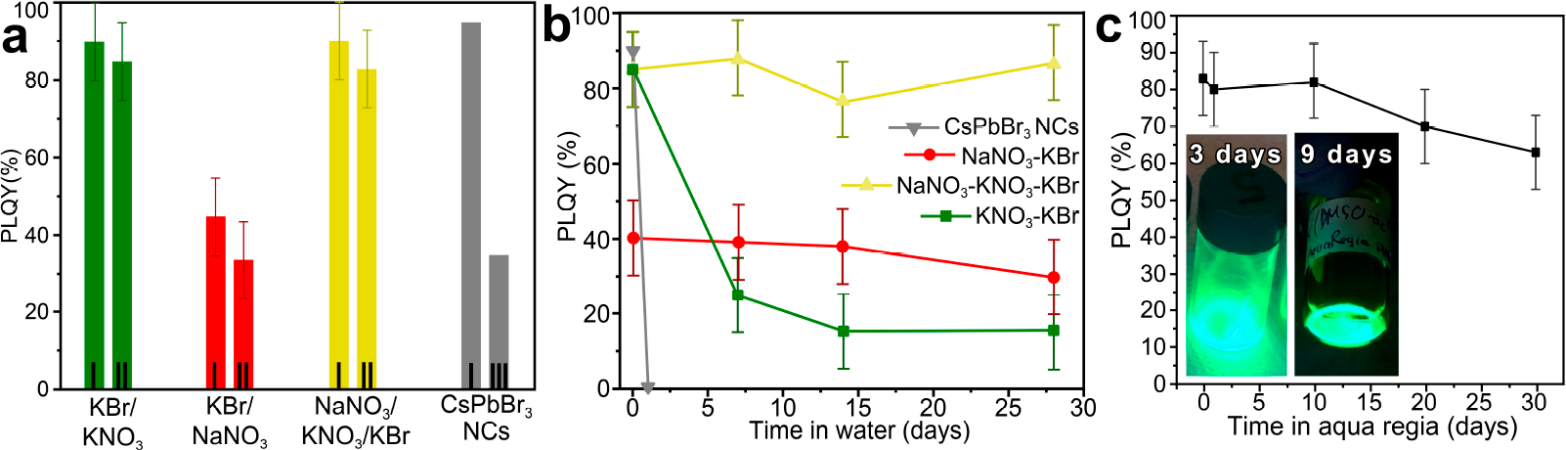
(a) PLQY of CsPbBr_3_/m-SiO_2_ composites that were prepared by employing different molten salts mixtures: KNO_3_–KBr, NaNO_3_–KBr, and KNO_3_–NaNO_3_–KBr and “standard” colloidal CsPbBr_3_ NCs, before (I) and after annealing at 180 °C in argon for (III) 2 h or (II) 3 h. (b) Time-dependent PLQY values of CsPbBr_3_/m-SiO_2_ composites and “standard” colloidal CsPbBr_3_ NCs immersed in water. (c) Time-dependent PLQY values of the CsPbBr_3_/m-SiO_2_ composite prepared with KNO_3_–NaNO_3_–KBr immersed in aqua regia (inset: photographs of CsPbBr_3_/m-SiO_2_ composite immersed in aqua regia for 3 and 9 days).

To reveal the morphology and the structure of our CsPbBr_3_/m-SiO_2_ composites, we performed an in-depth transmission electron microscopy (TEM) analysis. The starting m-SiO_2_ particles have a mean size of 0.6 μm, as emerged from our DLS measurements (Figure S2) and are characterized by pores arranged in a hexagonal framework, typical of MCM-41 silica, with the pores having a mean diameter of 3.3 nm (Figure S1b). Such mesoporous structure is only partially retained in the final composites whose TEM appearance indicates that the nanoparticles had grown inside the pores of SiO_2_, with the concomitant collapse of most of the pores (Figure 1b). To gain a deeper understanding of the nanostructure of the composites, we performed high-resolution (HR) TEM, high-angle annular dark-field (HAADF) scanning transmission electron microscopy (STEM), and energy-dispersive X-ray spectroscopy (EDS) analyses. These confirmed the partial collapse of the SiO_2_ mesoporous structure (Figure 1c, to be also compared with Figure 3a) and the formation of orthorhombic CsPbBr_3_ NCs inside the SiO_2_ particles (inset of Figure 1b), together with K, Na, and N-containing salts (with the K:Na ratio being close to 2:1) (Figure 1e), compatible with the XRD results.

In order to assess the stability of our composites, we exposed them to either high temperature (180 °C), to water, or to an acid + an oxidizing environment (aqua regia). Colloidal CsPbBr_3_ NCs prepared via a standard hot injection approach were also tested in parallel.^1^ The thermal stability tests were carried out by monitoring the variation of the PLQY of the sample before and after annealing at 180 °C for 3 h in argon atmosphere: the PLQY of our composite dropped from 89 to 85%, whereas that of colloidal CsPbBr_3_ NCs dropped from 90% to 30% after annealing at 180 °C in argon for 2 h (Figure 2a). The stability against water was assessed by dispersing and stirring the samples in DI water and monitoring the resulting PLQY over time. As shown in Figure 2b, the CsPbBr_3_/m-SiO_2_ composite was stable in water for 30 days with no visible drop in PL emission intensity, while the colloidal CsPbBr_3_ NCs degraded quickly, with a complete quenching of the PL emission after only a few minutes. Most notably, our composite was stable when immersed in aqua regia for 30 days (Figure 2c). The decay in PLQY was not accompanied by any notable shift in the spectral position of the PL, not even after 70 days of immersion in aqua regia (Figure S3). Overall, these stability tests highlight the high stability of the CsPbBr_3_/m-SiO_2_ composite that stems from the complete embedment of the LHP NCs inside SiO_2_. This is to be compared with previous works, in which CsPbBr_3_ NCs had been grown inside m-SiO_2_ without the use of molten salts and the resulting compounds could not even sustain a washing step with water or with other polar solvents (Table 1).^22−26,28^

**Figure 3:**
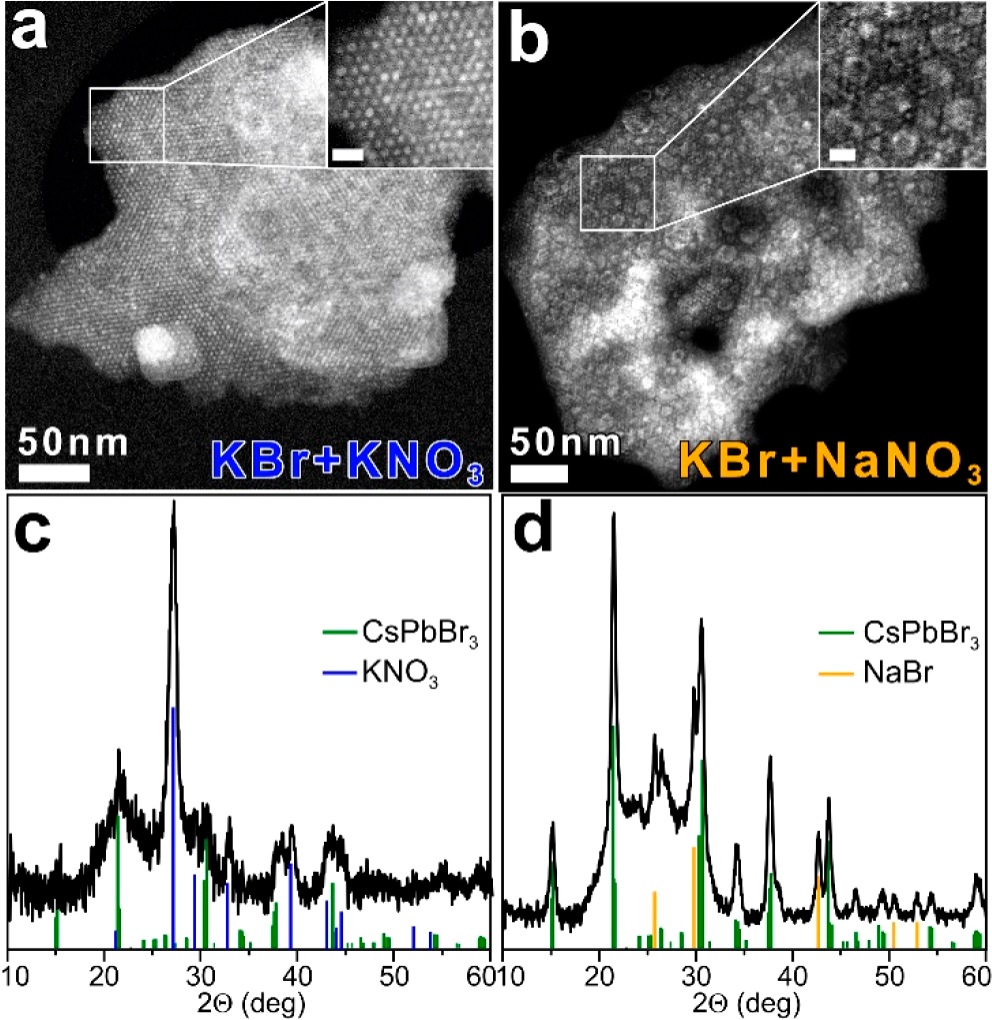
HAADF-STEM images and XRD patterns of the composites obtained by using KBr+KNO_3_ or KBr+NaNO_3_ molten salts mixtures (a,c) and (b,d), respectively. The scale bars in both insets are 10 nm. The bulk reflections of KNO_3_ (ICSD 98-003-6113), NaBr (ICSD 98-004-1440), and CsPbBr_3_ (ICSD 98-009-7851) are also reported by means of vertical bars.

On the other hand, the stability of our samples is comparable to that observed by Zhang. et al., who prepared CsPbBr_3_/m-SiO_2_ composites via a solid state reaction in which CsBr, PbBr_2_, and m-SiO_2_ were annealed together at high temperatures (Table 1).^27^ In their case the collapse of the mesoporous form of silica was observed only when working above 700 °C and, therefore, was attributed to high reaction temperatures, which also led to the merging of SiO_2_ particles. Their resulting heavily sintered/aggregated composites had a reduced PLQY (63%) that could only be moderately increased to 71% by an HF treatment. Conversely, as demonstrated by DLS measurements, our molten salts synthesis does not lead to merging or aggregation of the CsPbBr_3_/m-SiO_2_ particles (Figure S2), whose PLQY, being already very high, makes it unnecessary to perform further (and possibly hazardous) treatments.

**Table 1:** Comparison of the Different LHP/m-SiO_2_ Composites Reported in the Literature

				stability		
ref	reaction temperature	use of solvents	PLQY	water	acid	thermal
Chen et al.^22^	180 °C	yes	68%	Degradation and change in color after 15 min	N/A	N/A
Zhang et al.^27^	700 °C	no	63%	∼100% PL retention after 50 days	∼100% PL retention after 50 days	N/A
Wang et al.^26^	RT	yes	≤55%	N/A	N/A	∼95% PL retention after 1 cycle at 100 °C
Dirin et al.^23^	150 °C	yes	48%	N/A (degradation if the sample is cleaned with polar solvents)	N/A	N/A
Malgras et al.^25^	95 °C	yes	≤5.5%	N/A	N/A	N/A
this work	350 °C	no	90%	∼95% PL retention after 30 days	∼55% PL retention after 30 days	∼90% PL retention after 3h at 180 °C

The pore collapse and sealing in our nanocomposites can be tentatively explained by the corrosiveness of alkali salts to various metal oxides, which has been known for decades.^45,46^ In fact, molten salts have been even used to produce mesoporous structures starting from nonporous metal oxides,^45^ and, in particular, from silica.^42,47−50^ To better understand the role of molten salts on our final composites, we performed a series of control experiments in which we systematically varied the molten salts composition and investigated the structural and optical properties of the corresponding products. When the synthesis was performed with KBr and KNO_3_, the product, consisting of m-SiO_2_ particles whose pores are filled with LHP NCs and KNO_3_ (Figure 3a,c), exhibited a high PLQY (89 ± 10%) and a low resistance against water and aqua regia (Figure 2a,b).^51^ Interestingly, this procedure did not affect the mesoporous structure of m-SiO_2_ which was completely retained in the composite (Figure 3a). Conversely, the use of NaNO_3_ and KBr yielded composites having a low PLQY (42 ± 10%) and a high resistance against water and acid treatment (Figure 2a,b and Figure S4). The XRD and TEM analyses revealed that the product consisted of m-SiO_2_ particles filled with CsPbBr_3_ NCs and NaBr, which had partially lost their mesoporous structure (Figure 3b, d).

Overall, these control experiments indicate that the composition of the molten salts mixture employed has a profound impact on the structure of the final composites: (i) the use of NaNO_3_ is responsible for the partial collapse of the porous structure of the m-SiO_2_ particles as this salt is probably more corrosive than KNO_3_ and KBr toward silica; (ii) the presence of KNO_3_ inside SiO_2_ leads to an optimal PL emission of CsPbBr_3_ NCs, for reasons that are unclear at present. These observations suggest that a ternary mixture of KNO_3_, KBr, and NaNO_3_ is therefore essential to achieve both high PLQY and high stability, as experimentally observed by us. In another series of control experiments, in which we employed the ternary KNO_3_–KBr−NaNO_3_ molten salts mixture and then systematically varied their relative composition, we also observed that the resulting emitting composites were stable in aqua regia (hence the LHP NCs were completely embedded inside the SiO_2_ particles) only when working with KNO_3_:NaNO_3_:KBr ratios of 10:5:5, 8:7:5, 7:8:5, and 5:10:5 (Table S1 and Figure S4), with the first ratio also maximizing the PL emission of the product. These results indicate that both the composition and the stoichiometry of the molten salts mixture is of paramount importance in regulating the properties of the final composites.

Motivated by the optimal properties of our composites, we tested them in down converting LED (both on-chip and remote applications were tested). We also performed preliminary tests under conditions that are typical for oil tracing in the crude oil extraction industry, the latter requiring stability under high salinity conditions. For the fabrication of a white LED (on-chip application), a blue emitting LED (3 W, 3.2–3.4 V and wavelength: 445–450 nm) was covered by a mixture of our CsPbBr_3_/m-SiO_2_ composite (green emitting), K_2_SiF_6_:Mn (red emitting) powder, and TiO_2_ (light scattering agent) dispersed in poly(dimethylsiloxane). The white light emitted by the final device had CIE color coordinates of (0.2985, 0.3076) and a correlated color temperature (CCT) of 7692 K (Figure 4a,b). Being characterized by three distinct narrow emission peaks of blue, green, and red colors (Figure 4a), such white LED is promising as a light source in LCD backlighting for wider color gamut displays.^52^ In this regard, at present, the most used architecture for the backlighting unit in LCD displays consists of a blue LED light source (450–460 nm wavelength, as the backlight) and a color-converting polymer film containing green and red emissive quantum dots or phosphors (QDs-polymer composite films). In this configuration (remote application), part of the blue light is transmitted, and the rest is converted by the quantum dots/phosphor into green and red yielding a final white emission. In order to test if our composites would be efficient in such configuration, we prepared a mixture of CsPbBr_3_/m-SiO_2_, K_2_SiF_6_:Mn, and TiO_2_ powders, which was enclosed in a UV-curable acrylate polymer (isobornyl acrylate based) and sandwiched in between two transparent barrier polymer films, forming a (CsPbBr_3_/m-SiO_2_)-polymer film which was placed remotely from a blue emissive LED chip (450 nm wavelength and intensity of 200 mW/cm^2^) (Figure 4c). Compared to a standard CsPbBr_3_ NCs-polymer composite film, our (CsPbBr_3_/m-SiO_2_)-polymer film showed a superior stability, fully retaining the initial luminescence after a prolonged test of 240 h under high irradiation flux (Figure 4c). The (CsPbBr_3_/m-SiO_2_)-polymer film also exhibited high stability under ambient conditions, as shown by the absence of edge ingress (degradation of the NCs on the film edges where barrier the film is absent) that is typically observed at the edge of CsPbBr_3_ NCs-polymer films (Figure S5). The CsPbBr_3_/m-SiO_2_-polymer film obtained here gave a color point at *x* = 0.08992; *y* = 0.76927 according to the CIE 1931 color diagram. Utilizing this CsPbBr_3_/m-SiO_2_ as a green color emitter in down converter LCD application could cover 87% of Rec.2020 area (Figure 4d). These features make our composites particularly promising as green phosphors not only for down-converter film application in lighting but also in LCD applications. Indeed, commercially available green emissive phosphors have a very broad emission spectrum limiting the color gamut of displays.

**Figure 4:**
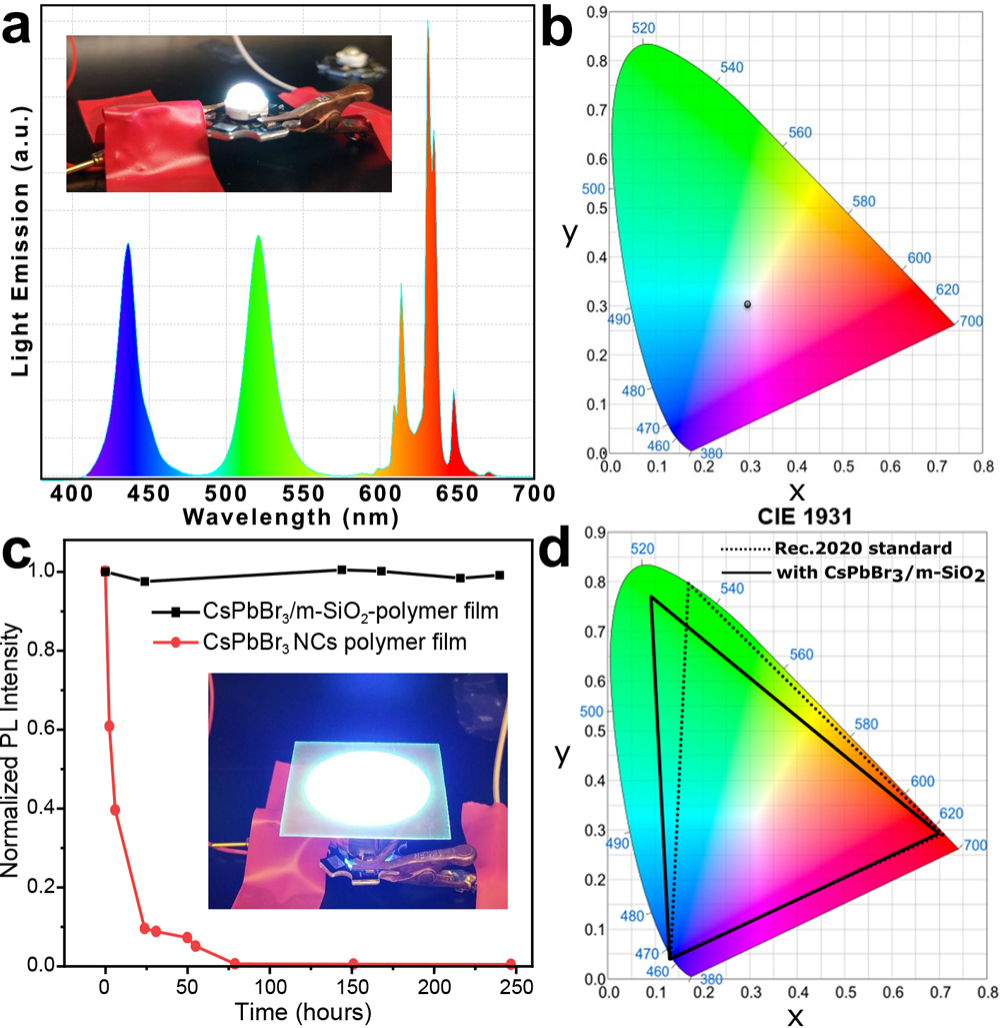
(a) Emission spectrum of the fabricated W-LED (insets: photograph of the W-LED under operation) and the corresponding (b) CIE1931 color coordinate diagram. (c) Time-dependent normalized PL intensity of CsPbBr_3_/m-SiO_2_ polymer composites film and standard CsPbBr_3_ NCs-polymer composite film under high flux remote application test (inset: photograph of the (CsPbBr_3_/m-SiO_2_)-polymer film with blue LED chip (200 mW/cm^2^) under operation). (d) Color coverage of CsPbBr_3_ NCs-polymer composite film as compared to the standard Rec.2020 area.

The CsPbBr_3_/m-SiO_2_ composite was also tested under very harsh conditions, including high temperature and salinity, which characterize the underground conditions that tracers, used in oil extraction wells, have to withstand.^53,54^ Such tracers are employed in order to probe the efficiency of injection wells drilled for oil extraction. To do so, tracers are inserted in the injection well, and their presence is probed at the extraction well: an amount of recovered tracer at the extraction well and the speed at which the tracer travels through the well can give precious information about the efficiency of the injection well.^41^ The PLQY of our composite was 83% after 24 h of incubation at RT in an aqueous solution of NaCl, CaCl_2_, MgCl_2_, Na_2_SO_4_, and NaHCO_3_, and 18% after an incubation of 168 h at 90 °C (Figure S6). In this last case, the observed decrease in PLQY was attributed to the aggregation of the CsPbBr_3_/m-SiO_2_ particles whose hydrodynamic radius, measured by DLS, increased from 0.6 μm (prior the test) to 10 μm after the test completion (Figure S6). Such radius could not be decreased even after sonication of the sample. These preliminary results indicate that our composites are potential candidates for oil tagging applications, but further improvements are needed to limit their aggregation: one way of solving this issue could be a surface functionalization of the composite with suitable molecules.

In conclusion, we have developed a molten salt synthesis route to prepare composites made of CsPbBr_3_ NCs embedded, together with inorganic salts (KNO_3_–NaNO_3_–KBr), in mesoporous SiO_2_ particles. The use of molten salts delivers composites with high PLQY (89 ± 10%) and, at the same time, for the sealing of SiO_2_ pores, thus conferring a high stability to the system: the CsPbBr_3_/m-SiO_2_ composites are resistant to heat, water, and even to aqua regia. Notably, such optical and physical properties were found to be intimately linked to the composition and stoichiometry of the molten salts employed: while NaNO_3_ is the main responsible of the sealing of the pores (conferring stability), KBr and KNO_3_ yield LHP NCs with a bright PL. Thanks to their optical and physical properties, our composites were found to be promising as green emitting phosphors in LEDs: the resulting device emitted white light with CIE color coordinates of (0.2985, 0.3076), CCT of 7692 K and exhibited a highly stable PL emission (in terms of peak position and intensity) after 240 h of operation. Also, pending further improvements in their stability, the composites should be suitable candidates as tracers for the oil industry, as they retain their PL emission after being exposed to high salinity at 90 °C for 7 days. We believe that this simple method can be extended to materials such as CsPbCl_3_ or CsPbI_3_ in order to get multicolor emission, as also indicated by our preliminary results in this direction (see Figure S7), and possibly, the method could also be used for Pb-free double perovskites.

## Supporting Information Available

Experimental Section, characterization of m-SiO_2_, DLS measurements, PL curves after the aqua regia treatment, composites synthesized by varying the KNO_3_:NaNO_3_:KBr molar ratio, tests of films employed for 10 days in remote configuration with a blue LED chip, test of composites exposed to brine, preparation of CsPb(Cl,Br)_3_ and CsPb(Br,I)_3_ based composites (PDF)
