# Supplementary material for: Low-Temperature Molten Salts Synthesis: CsPbBr3 Nanocrystals with High Photoluminescence Emission Buried in Mesoporous SiO2
Source: ACS Energy Lett. 2021 Feb 11;6(3):900–7. doi: 10.1021/acsenergylett.1c00052 (PMC8025713; doi:10.1021/acsenergylett.1c00052)
Supplement: Supplementary file 1 — nz1c00052_si_001.pdf [file nz1c00052_si_001.pdf]

# Low Temperature Molten Salts Synthesis: $CsPbBr_3$ Nanocrystals with High Photoluminescence Emission Buried in Mesoporous $SiO_2$

Mai Ngoc An[‡a,b], Sungwook Park[‡b,c*], Rosaria Brescia[d], Marat Lutfullin[e], Lutfan Sinatra[e], Osman Bakr[e,f], Luca De Trizio[b*] and Liberato Manna[b*]

[a] Dipartimento di Chimica e Chimica Industriale, Università degli Studi di Genova, Via Dodecaneso 31, 16146 Genova, Italy

[b] Nanochemistry Department and [d]Electron Microscopy Facility, Istituto Italiano di Tecnologia, Via Morego 30, 16163 Genova, Italy

[c] Department of Energy Science and Center for Artificial Atoms, Sungkyunkwan University, Suwon, 16419, Republic of Korea

[e] Quantum Solutions, 1 Venture Road, Southampton Science Park, SO16 7NP. UK (www.qdot.inc)

[f] Division of Physical Sciences and Engineering, King Abdullah University of Science and Technology (KAUST), Thuwal, 23955-6900, Saudi Arabia

## EXPERIMENTAL SECTION

***Chemicals.*** Lead bromide ($PbBr_2$, 99.999% trace metals basis), Cesium bromide (CsBr, 99.999% trace metals basis), Potassium nitrate ($KNO_3$, 99.999% trace metals basis), Sodium nitrate ($NaNO_3$, 99.995% trace metals basis), Potassium bromide (KBr, 99.99% trace metals basis), Silica (mesostructured, MCM-41 type, product code 643645), Dimethyl sulfoxide (DMSO) and Poly(dimethylsiloxane) (PDMS) were purchased from Sigma-Aldrich and used as received.

***Synthesis of $CsPbBr_3$/m-$SiO_2$ composites.*** A mixture of CsBr (1 mmol), $PbBr_2$ (1 mmol) and molten salts (see below for details) and commercial MCM-41 m-$SiO_2$ (5 mmol) was prepared by grinding with a mortar and pestle, transferred into a ceramic crucible and heated up to 350 °C in a furnace for 60 minutes under air. The resulting product was allowed to naturally cool down to room temperature (RT) and washed with a polar solvent such as DMSO or DMF or even with deionized (DI) water. Washing with DI water however causes the co-precipitation of PbBrOH and was therefore not considered further in this work. In a typical washing procedure, 100 mg of as-prepared powder was loaded in a vial with 1 ml of DMSO (or DMF) and sonicated for 10 minutes, centrifuged at 5000 rpm, and the supernatant was discarded. This procedure was repeated 5 times in order to quantitatively remove all the inorganic salts and $CsPbBr_3$ crystals that grown outside of the m-$SiO_2$ particles. The final powder was eventually dried in a vacuum oven at 40 °C. Different products were prepared by employing either a binary or ternary mixtures of molten salts: KBr:$KNO_3$ and KBr:$NaNO_3$ in a 5:15 mmol ratio; $KNO_3$:$NaNO_3$:KBr in 10:5:5 mmol ratios.

***Optical Measurements.*** The UV–vis absorption spectra were recorded using a Varian Cary 5000 UV–Vis-NIR spectrophotometer equipped with an integrating sphere. The PL spectra were measured on a Varian Cary Eclipse spectrophotometer using an excitation wavelength of 350 nm. The samples were prepared by dispersing the composite powders in hexane followed by drop-casting onto a quartz substrate. PLQY measurements were performed

using Fluorescence Spectrometer FS5 Edinburgh Instruments equipped with an integrating sphere. Measurement of LED color position, CRI and CCT parameters were performed with GL Spectis 5.0 touch-GL Optic spectrometers.

***Powder X-ray Diffraction (XRD).*** XRD patterns were acquired on a PAN analytical Empyrean X-ray diffractometer, equipped with a 1.8 kW Cu Kα ceramic X-ray tube and a PIXcel3D 2 × 2 area detector, operating at 45 kV and 40 mA, under ambient conditions using parallel beam geometry and symmetric reflection mode. Samples were prepared by pressing the composite powders onto a quartz zero-diffraction single crystal substrate.

***Dinamic Light Scattering (DLS) measurements.*** DLS measurements were performed by using a Zetasizer Nano S from Malvern Pananalytical. The composite were dispersed in water or in the brine solution for the measurements. The refractive index of the $CsPbBr_3$/m-$SiO_2$ composite were taken from the work of Yan et al..[1]

***Transmission Electron Microscopy (TEM).*** Overview bright-field TEM (BF) analyses were performed on a JEOL JEM-1011 TEM (W filament) operated at 100 kV. High-resolution TEM (HRTEM) and high angle annular dark-field scanning TEM (HAADF-STEM) imaging were carried out on a JEOL JEM-2200FS TEM (Schottky emitter), operated at 200 kV, equipped with a CEOS corrector for the objective lens and an in-column image filter (Ω-type). Due to the fast beam damage undergone by these particles, they were exposed to a relatively low dose rate (~ 30 electrons/($Å^2$ s)) and HRTEM images were acquired using a direct electron detection camera (K2 Summit, Gatan), in super-resolution mode. Each image shown here is obtained from a $(260\ nm)^2$ frame obtained by summing aligned frames obtained by short exposure (0.4 s), with a total acquisition time of 12 s. STEM- Energy-dispersive X-ray spectroscopy (EDS) data were acquired in STEM mode by a Bruker XFlash 5060 silicon-drift detector installed on the same microscope. Samples for HRTEM, HAADF-STEM and STEM-EDS mapping were prepared by mildly sonicating the composite powder suspended in ethanol and drop-casting of the suspension supernatant onto a holey carbon film on Cu. The STEM-EDS maps were obtained by simple integration, point by point in the maps, of the K peaks of N, O and Na, the Kα peaks of Si, Na, K and Br, and the Lα peaks of Pb and Cs.

***LED on-chip device fabrication.*** For the fabrication of a white LED (on-chip application), a mixture of $CsPbBr_3$/m-$SiO_2$ composite (green emitting), $K_2SiF_6$:Mn (red emitting) powder ,$TiO_2$ (light scattering agent) and PDMS-poly(dimethylsiloxane) was prepared. The wt% ratio of $CsPbBr_3$/m-$SiO_2$ and $K_2SiF_6$:Mn is 1:4. The mixture was deposited onto a blue LED chip (3 watts, 3.2-3.4 V and wavelength of 445-450nm) and heated in the oven at 60°C for 30 minutes.

***$CsPbBr_3$/m-$SiO_2$-polymer composite film preparation.*** For the fabrication of $CsPbBr_3$/m-$SiO_2$-polymer composite films, the $CsPbBr_3$/m-$SiO_2$ composite (green emitting) powder was mixed with $K_2SiF_6$:Mn (red emitting) powder,$TiO_2$ (light scattering agent) and a UV-curable polymer (isobornyl acrylate based). The wt% ratio of $CsPbBr_3$/m-$SiO_2$ and $K_2SiF_6$:Mn is 1:4. The mixture was deposited in between 3M transparent barrier films using blade coating and cured under high flux UV lamp for 30 seconds.

***Stability tests for oil tracing applications.*** The stability measurements for oil tracing applications were performed in water and brine solutions. The brine composition is the following: 150.446 g of NaCl, 69.841 g of $CaCl_2$, 20.396 g of $MgCl_2$, 0.518 g of $Na_2SO_4$ and 0.487 g of $NaHCO_3$ dissolved in 1lt of distilled water. In this study, the $CsPbBr_3$/m-$SiO_2$ were tested in 3 different conditions: water at RT for 24h, brine solution at RT for 24h and brine solution at 90°C for 24h. $CsPbBr_3$/m-$SiO_2$ were dispersed in water/brine solution with a concentration of 1 mg/mL. The size and PL intensity of the composites were compared before and after each test.

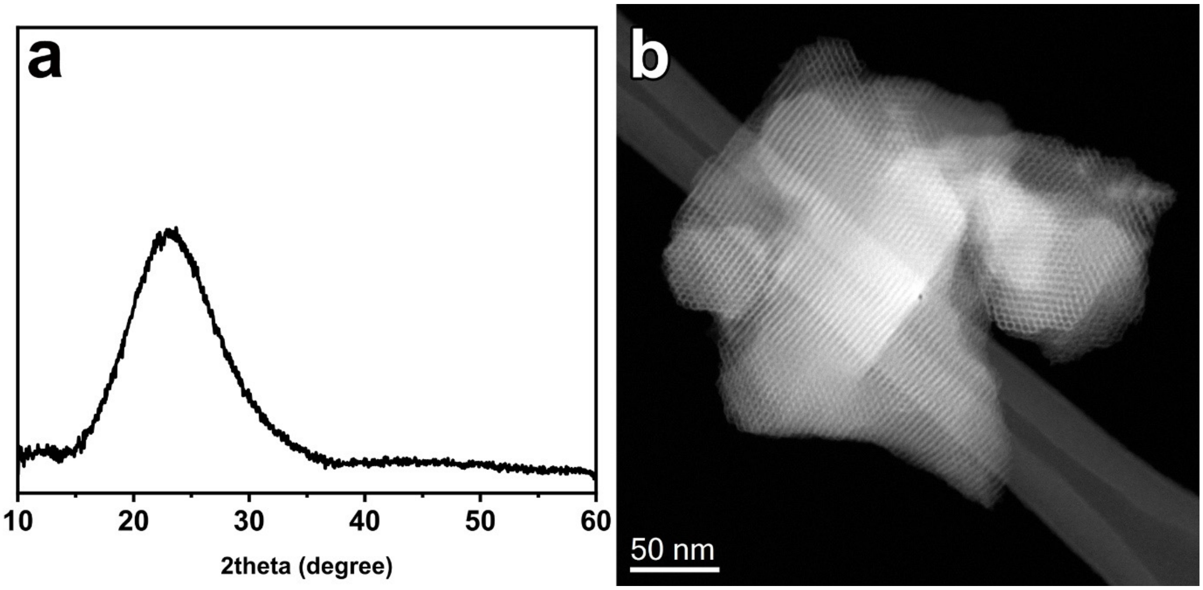


**Figure S1.** (a) XRD pattern of starting m-$SiO_2$ MCM 41 (Sigma Aldrich code 643645) showing a broad peak ranging from 15° to 35°, which is ascribed to the amorphous $SiO_2$ matrix. (b) HAADF-STEM image of the starting MCM-41 m-$SiO_2$.

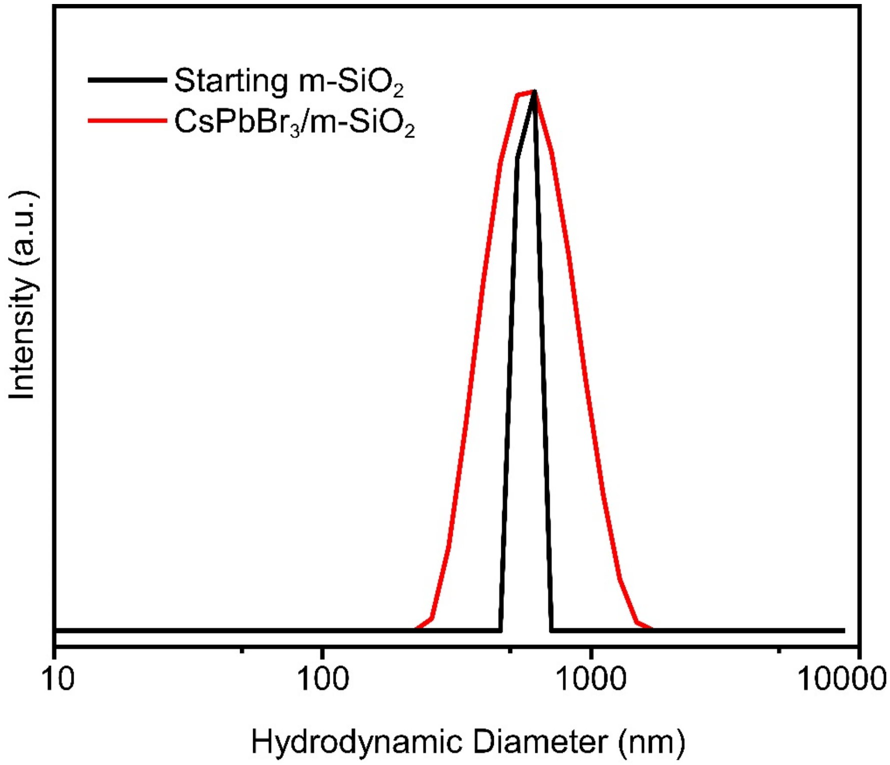


**Figure S2**. DLS curves obtained for starting m-$SiO_2$ particles and $CsPbBr_3$/m-$SiO_2$ composites dispersed in water.

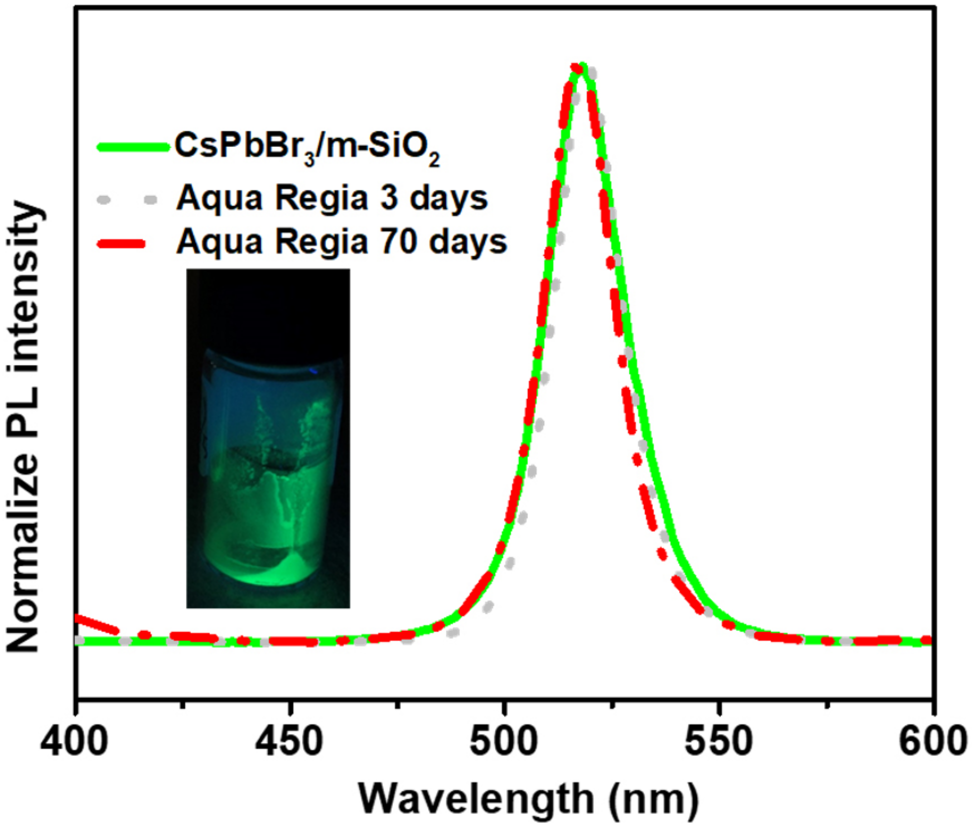


**Figure S3.** PL spectra of $CsPbBr_3$/m-$SiO_2$ composite before (green curve) and after immersion in aqua regia for 3 days (gray curve) and 70 days (red curve). The intensity of the PL peaks has been adjusted in order to better show that the peak position did not change during this test. Inset: Photograph of $CsPbBr_3$/m-$SiO_2$ composite immersed in aqua regia for 70 days under UV light (345 nm).

**Table S1. Molar ratio of $KNO_3$:$NaNO_3$:KBr in different mixtures for $CsPbBr_3$/m-$SiO_2$ composites synthesis**

| Sample | 0 | 1 | 2 | 3 | 4 | 5 | 6 | 7 | 8 | 9 | 10 | 11 | 12 | 13 |
|---|---|---|---|---|---|---|---|---|---|---|---|---|---|---|
| $KNO_3$ (mmol) | 15 | 14 | 13 | 12 | 11 | 10 | 7.5 | 9 | 8 | 7 | 6 | 5 | 4 | 0 |
| $NaNO_3$ (mmol) | 0 | 1 | 2 | 3 | 4 | 5 | 7.5 | 6 | 7 | 8 | 9 | 10 | 11 | 15 |
| KBr (mmol) | 5 | 5 | 5 | 5 | 5 | 5 | 5 | 5 | 5 | 5 | 5 | 5 | 5 | 5 |

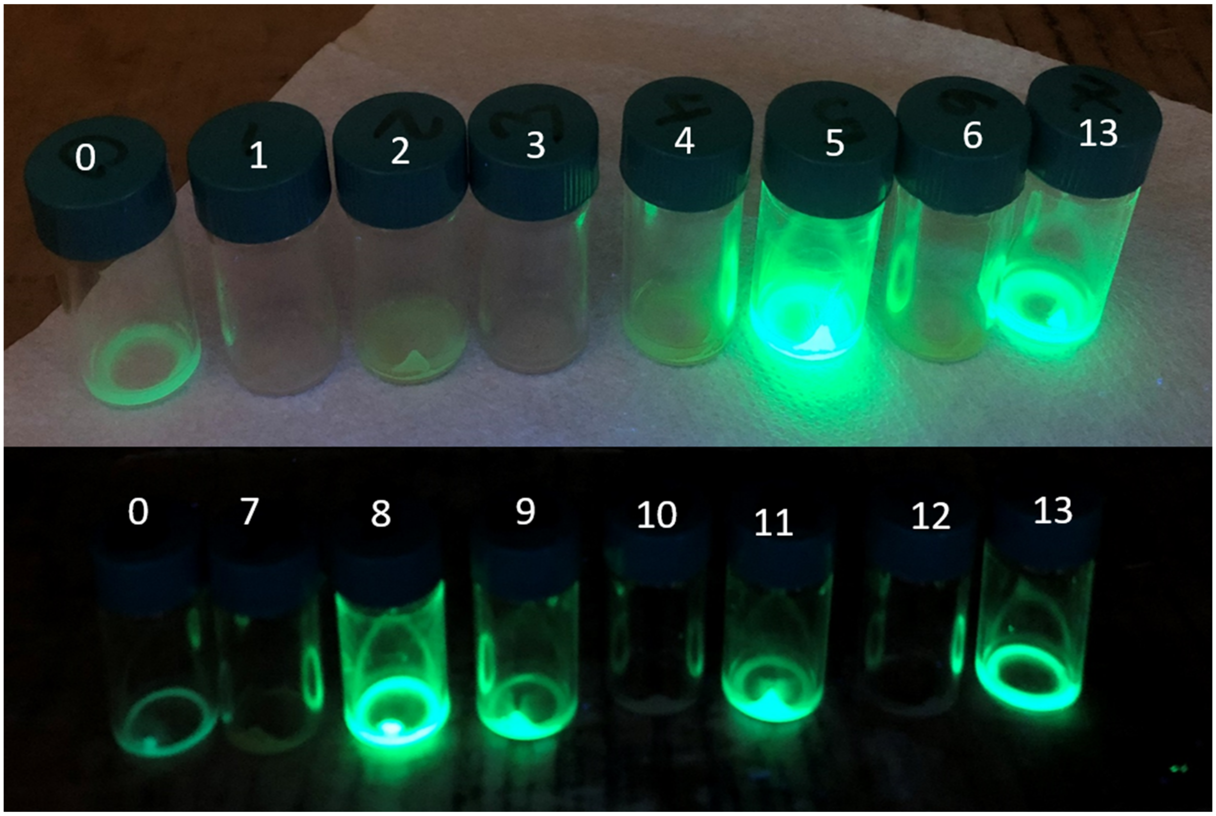


**Figure S4.** Photographs taken under UV light illumination (345 nm) of $CsPbBr_3$/m-$SiO_2$ composites synthesized by varying the $KNO_3$:$NaNO_3$:KBr molar ratio (see Table S1), after being immersed in aqua regia for 3 days.

**After 10 days remote application on LED chip:**

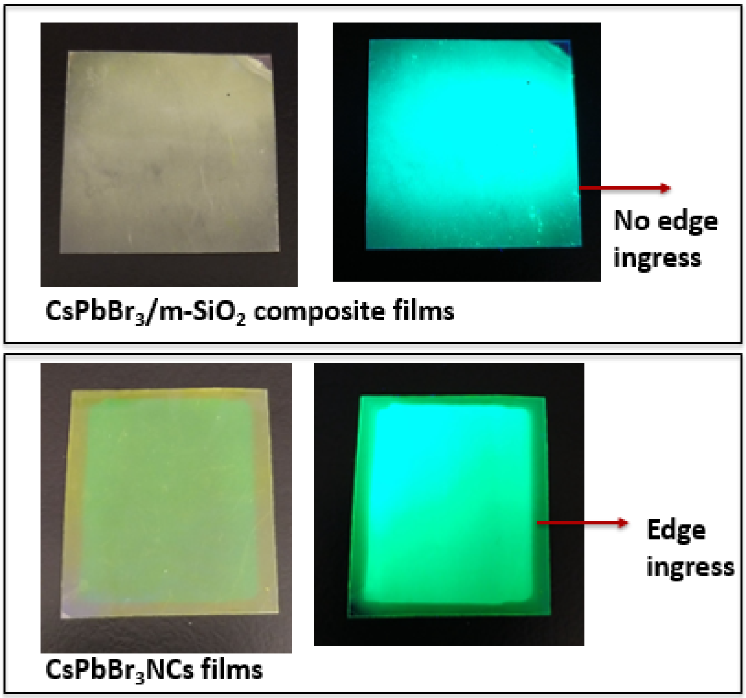


**Figure S5.** Photographs taken under visible (left) and UV light (right) of: (upper panel) a ($CsPbBr_3/m-SiO_2$)-polymer film which does not show any edge ingress after being tested for 10 days in remote configuration with a blue LED chip (200 mW/$cm^2$); (lower panel) a $CsPbBr_3$ NCs-polymer film (the $CsPbBr_3$ NCs were synthesized by following the standard colloidal method of Protesescu et al.[2]) tested for 10 days in remote configuration with a blue LED chip (200 mW/$cm^2$) in which it is possible to observe the appearance of the edge ingress.

Comparison PLQY and PL Intensity:

| | In water | In brine 24h | In brine 24h follow by heat 90C in brine 24h |
|---|---|---|---|
| PLQY | 89% | 74% | 18% |
| PL Peak Intensity | $6.8 \times 10^6$ | $7.0 \times 10^5$ | $3.5 \times 10^5$ |
| photo | 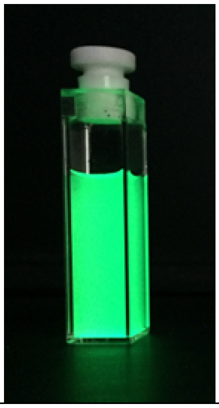 | 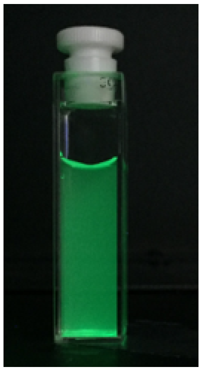 | 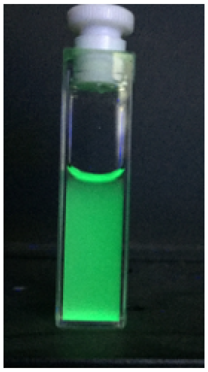 |

Particle size summary

| | In water | In brine 24h | In brine 24h follow by heat 90C in brine 24h |
|---|---|---|---|
| Particle size | ~ 0.6 microns | ~ 1.1 microns | ➢ 10 microns |

**Figure S6.** PLQY and size distribution (measured by DLS) of $CsPbBr_3$/m-$SiO_2$ composites before and after the immersion in brine for 24h at either RT or 90°C.

Synthesis procedure of $CsPb(Br,I)_3$ and $CsPb(Cl,Br)_3$ based composites:

$CsPb(Br,I)_3$-based composites were prepared by mixing CsBr/CsI (0.8 mmol/0.2 mmol), $PbBr_2/PbI_2$ (0.8 mmol/0.2 mmol), KBr/KI (4 mmol/1 mmol), $KNO_3$ (15 mmol) and m-$SiO_2$ (5 mmol). The mixture was heated in a crucible at 350 °C for 60 minutes under air and then it was allowed to cool down to room temperature.

$CsPb(Cl,Br)_3$-based composites were prepared by mixing CsCl/CsBr, $PbCl_2/PbBr_2$, KCl/KBr, $KNO_3$ and m-$SiO_2$ (see detail ratio in Table 1). The mixture was heated in a crucible at 350 °C for 60 minutes under air. After cooling down to room temperature, the final product was washed with distilled water and dried in a vacuum oven at 40 °C.

**Table 1. Preparation conditions of CsPb(Cl,Br)3-based nanocomposites.**

| No. | PL peak | Perovskite Precursors ratio | | Molten salts ratio | | Silica | Reaction | |
|---|---|---|---|---|---|---|---|---|
| | | CsBr:CsCl | $PbBr_2$:$PbCl_2$ | KBr:KCl | $KNO_3$ | $SiO_2$ | Temperature | Time |
| 1 | 520 nm | 1:0 | 1:0 | 5:0 | 15 | 5 | 350 °C | 60 min |
| 2 | 510 nm | 1:0 | 0.33:0.67 | 5:0 | | | | |
| 3 | 505 nm | 1:0 | 0.17:0.83 | 5:0 | | | | |
| 4 | 500 nm | 1:0 | 0.11:0.89 | 5:0 | | | | |
| 5 | 487 nm | 0:1 | 0:1 | 5:0 | | | | |
| 6 | 461 nm | 0:1 | 0:1 | 4:1 | | | | |
| 7 | 443 nm | 0:1 | 0:1 | 2.5:2.5 | | | | |

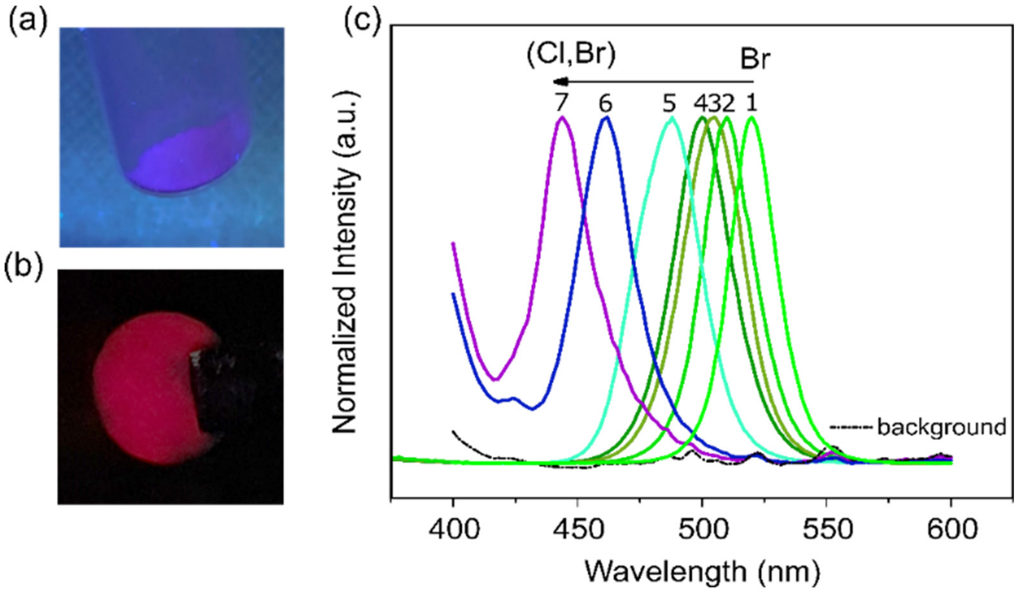


**Figure S7.** Photographs of (a) $CsPb(Cl,Br)_3$ and (b) $CsPb(Br,I)_3$ based nanocomposites under UV illumination at 365 nm; (c) PL spectra of $CsPb(Cl,Br)_3$ based composites with the PL tunable from 520 nm (curve 1) to 443 nm (curve 7) by varying the halide composition.

REFERENCES

(1) Yan, W.; Mao, L.; Zhao, P.; Mertens, A.; Dottermusch, S.; Hu, H.; Jin, Z.; Richards, B. S., Determination of Complex Optical Constants and Photovoltaic Device Design of All-Inorganic $CsPbBr_3$ Perovskite Thin Films. *Opt. Express* **2020,** *28*, 15706-15717.

(2) Protesescu, L.; Yakunin, S.; Bodnarchuk, M. I.; Krieg, F.; Caputo, R.; Hendon, C. H.; Yang, R. X.; Walsh, A.; Kovalenko, M. V., Nanocrystals of Cesium Lead Halide Perovskites ($CsPbX_3$, X = Cl, Br, and I): Novel Optoelectronic Materials Showing Bright Emission with Wide Color Gamut. *Nano Lett.* **2015,** *15*, 3692-3696.
